# Sex Differences in Long-Term Outcomes of Left Atrial Appendage Closure—Analysis from the LEADER Registry

**DOI:** 10.3390/jcm15041604

**Published:** 2026-02-19

**Authors:** Aviad Rotholz, Hagai Itach, Roi Ferman, Tsahi T. Lerman, Avi Sabbag, Israel M. Barabash, Ehud Chorin, Roei Merin, Hana Vaknin Assa, Alexander Omelchenko, Aharon Erez, Gregory Golovchiner, Leor Perl, Ran Kornowski, Amos Levi

**Affiliations:** 1 Cardiology Department, Beilinson Hospital, Rabin Medical Center, Ze’ev Jabotinsky St 39, Petah Tikva 4941492, Israel; 2 Gray Faculty of Medical and Health Sciences, Tel Aviv University, P.O. Box 39040, Ramat Aviv, Tel Aviv 6997801, Israel; 3 The Adelson School of Medicine at Ariel University, 3 Kiryat Hamada St., Ariel 4070000, Israel; 4 Cardiology Department, Sheba Medical Center, 2 Derech Sheba, Ramat Gan 5262100, Israel; 5 Cardiology Department, Tel Aviv Sourasky Medical Center (Ichilov), 6 Weizmann St., Tel Aviv-Yafo 6423906, Israel; 6 Cardiology Department, Meir Medical Center, 59 Tchernichovsky St., Kfar Saba 4428164, Israel

**Keywords:** LAAC, sex differences, outcomes

## Abstract

**Background:** Percutaneous left atrial appendage closure (LAAC) provides an alternative to oral anticoagulation (OAC) in atrial fibrillation (AF) patients who are at high bleeding risk. Prior studies have suggested sex-related differences in procedural outcomes, with women demonstrating higher peri-procedural complication rates. Data on long-term outcomes, however, remain inconsistent. **Methods:** We analyzed 407 consecutive patients with AF who underwent LAAC between 2010 and 2023 in four Israeli medical centers participating in the LEADER registry. Baseline characteristics, procedural data, and clinical outcomes were compared between men and women. The primary efficacy endpoint was ischemic stroke or systemic embolism at 1 year. The primary safety endpoint was a composite of all-cause mortality, procedural complications, or major bleeding at 1 year. **Results:** Of 407 patients, 285 (70%) were men and 122 (30%) were women. The mean age was 77 ± 8.4 years with similar CHA_2_DS_2_-VASc and HAS-BLED scores across sexes. Device implantation exceeded 99% in both sexes. Major peri-procedural complications occurred in 6.4% overall, without significant sex-based differences (men 7.0%, women 4.9%, *p* = 0.51). At 1-year follow-up, Kaplan–Meier estimates for the primary efficacy endpoint of ischemic stroke/systemic embolism (2.6%), the primary safety endpoint (19.2%), major bleeding (8.9%), and all-cause mortality (9.3%) were comparable between men and women (all *p* > 0.1). **Conclusions:** In contrast to prior large registries reporting higher peri-procedural risk in women, this real-world multicenter experience demonstrated no significant sex differences in either peri-procedural or long-term outcomes following LAAC. These findings support LAAC as an effective and safe stroke-prevention strategy in AF, irrespective of sex.

## 1. Introduction

Atrial fibrillation (AF) is the most common sustained cardiac arrhythmia, affecting millions worldwide and imposing substantial individual and healthcare burdens through increased risks of stroke, heart failure, dementia, and mortality.

While men have a higher overall incidence and prevalence of AF, important sex-based differences exist in clinical presentation, risk profiles, and outcomes. Women with AF tend to be older at diagnosis, present with more severe symptoms and worse quality of life, and have higher rates of hypertension, valvular heart disease, and heart failure with preserved ejection fraction compared to men. Female sex serves as a stroke risk modifier in AF, conferring approximately 20–30% higher stroke risk, even after adjusting for other risk factors and anticoagulation therapy [1,2].

This excess stroke risk is particularly pronounced in women aged 75 years and older, with women experiencing not only higher stroke rates but also greater stroke severity and more permanent disability following cerebrovascular events [3,4].

Percutaneous left atrial appendage closure (LAAC) has emerged as a minimally invasive alternative to OAC therapy in patients with atrial fibrillation at increased risk of stroke, aimed at reducing the bleeding risk associated with pharmacotherapy without paying a penalty of increased risk of thromboembolic events [5]. Emerging data suggest significant sex-based differences in both short-term procedural complications and potentially long-term outcomes. Multiple studies and meta-analyses have demonstrated that women experience higher rates of periprocedural complications, including pericardial effusion/tamponade (1.5–1.8-fold increased risk), major bleeding (1.3–2.0-fold increased risk), and vascular complications (1.4–1.8-fold increased risk) compared to men, despite similar procedural success rates [6,7,8]. Women also demonstrate higher rates of renal complications and in-hospital mortality following LAAC [9]. Data from the NCDR LAAO Registry has shown that when using the Watchman device, compared with men, women have a significantly higher risk of in-hospital adverse events after LAAC [10]. A Japanese cohort identified that women were more susceptible to pulmonary embolism after discontinuing anticoagulant therapy post-LAAC than men [11]. Interestingly, from a quality of life standpoint, based on an EQ-5D-3L questionnaire, women had a significant improvement in their health state as assessed several months after LAAC, compared to their male counterparts [12].

Regarding long-term outcomes after LAAC, the data are more conflicting: some studies such as the EWOLUTION registry report no significant sex differences in mortality, stroke, or thromboembolic events at 1–2 year follow-up, while others suggest women remain at 13–20% higher risk for readmissions at 30 days and 6 months post-procedure [6,7,8,13,14]. Analysis from the Italian-FLX registry showed comparable rates for all-cause mortality, stroke, transient ischemic attack and major bleeding in men and women 12 months after the procedure. Notably, no significant gender differences were found in that registry for periprocedural complications [15]. In a different long-term outcome analysis over 4.3 years, women had a lower rate of fatal or disabling ischemic stroke compared with men [16].

We herein present an Israeli multicenter experience of LAAC using the Watchman and Amplatzer devices for over a decade. Our aim was to compare sex differences in the short and long-term of up to 3 years follow-up, adding to the existing body of evidence in the field.

## 2. Materials and Methods

### 2.1. Study Design

Data were derived from the LEADER (Left Atrial Appendage Exclusion Collaboration in Israel) registry, a national, multicenter Israeli registry designed to evaluate the procedural characteristics, safety, and long-term outcomes of patients undergoing percutaneous left atrial appendage closure (LAAC) in routine clinical practice. Consecutive patients were prospectively entered by each participating center and uploaded anonymously into a dedicated online case report form (CRF), including demographic, clinical, imaging, procedural, and follow-up data.

Four tertiary centers in Israel participated in the registry: Rabin Medical Center (RMC), Chaim Sheba Medical Center, Tel Aviv Sourasky Medical Center, and Meir Medical Center. The registry was conducted in accordance with the principles of the Declaration of Helsinki. The study protocol was approved by the institutional ethics committees of all participating centers, and the requirement for informed consent was waived due to the retrospective analysis of anonymized data.

Procedural outcomes, clinical events, and device-related complications were defined and reported in accordance with contemporary consensus recommendations for LAAC studies, including the Munich consensus document, where applicable [17].

### 2.2. Study Population

All consecutive patients undergoing percutaneous LAAC at Rabin Medical Center, Chaim Sheba Medical Center, Tel Aviv Sourasky Medical Center and Meir Medical Center between 2010 and 2023 were included (Table S1 in the Supplementary Materials). There were no sex-based exclusion criteria.

The baseline characteristics were obtained from electronic medical records and included demographics, cardiovascular risk factors, comorbidities, prior thromboembolic and bleeding events, and indication for LAAC. Preprocedural imaging included transesophageal echocardiography (TEE) and/or cardiac computed tomography (CCT), performed to exclude left atrial appendage thrombus and to assess appendage anatomy.

Procedural details, periprocedural complications, and postprocedural antithrombotic therapy were recorded. Long-term follow-up data included all-cause mortality, ischemic stroke, systemic embolism, and bleeding events.

### 2.3. Procedures

All patients underwent preprocedural clinical evaluation by a cardiologist, including physical examination, laboratory testing, and cardiac imaging. LAAC was performed via femoral venous access with transseptal puncture under general anesthesia with TEE guidance or under conscious sedation with intracardiac echocardiography (ICE), according to operator and institutional preference. Device selection and implantation strategy were left to the discretion of the implanting physician.

Procedural outcomes were classified in accordance with Munich consensus definitions. Technical success was defined as successful deployment of the device in the intended position. Device success was defined as stable device implantation without device embolization and with acceptable residual peri-device flow at the end of the procedure. Procedural success was defined as device success in the absence of in-hospital major procedure-related complications.

The recorded periprocedural complications included pericardial effusion or tamponade requiring intervention, device embolization, procedure-related stroke or transient ischemic attack, major vascular access complications, and procedure-related death.

Follow-up cardiac imaging was performed as part of routine postprocedural care at the discretion of the treating center. TEE and/or CCT were used to evaluate the device position and to assess for device-related thrombosis (DRT) and peri-device leak (PDL). The presence or absence of DRT and PDL at the first available postprocedural imaging assessment was recorded. Quantitative grading of peri-device leak size was not uniformly available.

Postprocedural antithrombotic therapy was prescribed according to local institutional protocols and individual patient risk profiles and was reassessed during follow-up, typically within 30–90 days, incorporating clinical status, imaging findings, and thromboembolic and bleeding risk.

### 2.4. Study Outcomes

The primary efficacy endpoint was a composite of ischemic stroke or systemic embolism at 1-year follow-up. The primary safety endpoint was a composite of all-cause mortality, major procedural complications, or major bleeding at 1 year.

Secondary endpoints included periprocedural complications, all-cause mortality, ischemic stroke, systemic embolism, and major bleeding at 1, 2, and 3 years of follow-up. Device-related outcomes included device-related thrombosis and peri-device leaks detected on follow-up imaging.

Bleeding events were identified based on clinical documentation and hospitalization records and classified as major bleeding events according to clinical relevance, in alignment with consensus recommendations, although formal BARC subclassification was not available for all centers.

Clinical events were extracted from medical records at each participating center; no central adjudication committee was used.

### 2.5. Statistical Analysis

Baseline characteristics were compared between men and women. Continuous variables are presented as mean ± standard deviation or median with interquartile range, as appropriate. Categorical variables are reported as counts and percentages. Between-group comparisons were performed using Student’s *t*-test or the Wilcoxon rank-sum test for continuous variables and the χ^2^ test for categorical variables.

Time-to-event analyses for mortality, stroke, and bleeding outcomes were performed using Kaplan–Meier estimates and compared using the log-rank test. Cox proportional hazard regression models were constructed to evaluate sex-related differences in the primary efficacy and safety endpoints, as well as mortality, bleeding, and neurovascular events at 1 and 3 years. Covariate selection for multivariable analyses was guided by clinical relevance, based on the prior literature and expert judgment.

Potential predictors of procedural success were assessed using logistic regression, with the results reported as odds ratios and 95% confidence intervals.

Subgroup analyses were performed to evaluate the consistency of sex-related differences across clinically relevant subgroups, with interactions being assessed using multiplicative interaction terms.

To control for variance in the baseline characteristics, two-stage propensity score-matching was performed. A 1:1 cohort of men and women was constructed, using a 0.2 caliper and nearest neighbor matching. Matching quality was assessed by comparing standardized mean differences (SMD), with an SMD < 0.2 indicating acceptable balance. The balance of covariates before and after matching was visualized using Love plots.

All statistical tests were two-sided, and a *p*-value < 0.05 was considered statistically significant. Analyses were performed using R (RStudio, version 4.0.0; Vienna, Austria).

## 3. Results

**Figure 1:**
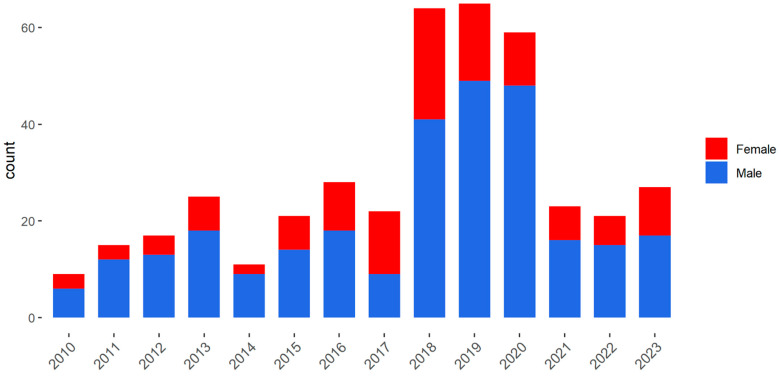
Temporal distribution of LAAC devices implanted between 2010 and 2023 in four medical centers in Israel: 285 men (70%) and 122 women (30%). Men are in blue and women are in red.

Our cohort included 407 consecutive patients with AF who underwent LAAC in four medical centers in Israel between 2010 and 2023. Site-based temporal distribution is displayed in Table S1 in the Supplementary Materials. Two hundred and eighty five were men (70%) and 122 were women (30%). Gender-based temporal distribution of LAAC procedures is shown in Figure 1. Implanted devices consisted mainly of newer generation platforms comprising 74.7% of the cohort (98 WatchmanFLX and 206 Amplatzer Amulate). No sex-related differences in platform use were detected (*p* = 0.11, Table S2 in the Supplementary Materials).

The mean age was 77 ± 8.4 years, without a significant difference between men and women. The mean CHADS2 score and CHA2DS2-VASc score were 3.1 ± 1.3 and 5.1 ± 1.6, respectively, and the mean HAS-BLED score was 3.9 ± 0.95, without significant differences between men and women. The most common comorbidities consisted of hypertension (86%), previous significant bleeding (84.7%), previous cerebrovascular disease (56.2%) and diabetes mellitus (45.6%), with similar rates between men and women. Men displayed increased rates of coronary artery disease compared to women (53.9% vs. 33.1%, *p* < 0.001) as well as overall reduced LV function (*p* = 0.001). Additional baseline characteristics and other comorbidities are listed in Table 1.

**Figure 2:**
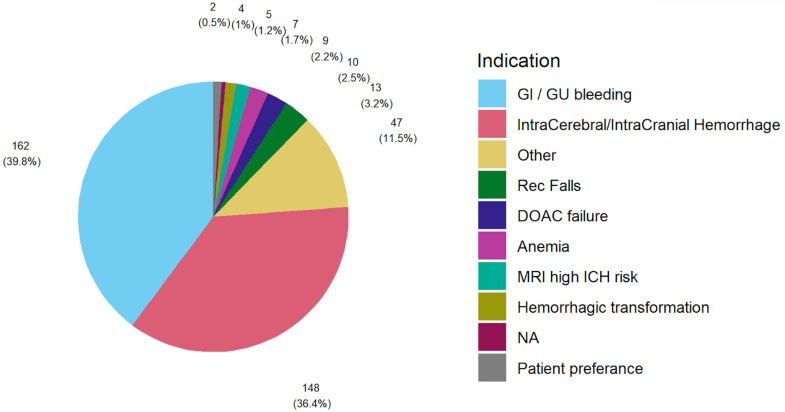
LAAC indications. The most common indications were gastrointestinal/genitourinary bleed (39.8%), followed by intracranial/intracerebral hemorrhage (36.4%).

LAAC was indicated as an alternative to anticoagulation, most commonly due to gastrointestinal/genitourinary bleeding (39.8%), followed by intracranial/intracerebral hemorrhage (36.4%). There were no significant differences in indications between men and women (*p* = 0.17). Additional indications of LAAC are specified in Figure 2 and Table S3 in the Supplementary Materials.

Baseline TEE was performed in 74.9% of patients and cardiac CT in 67%, with similar rates between men and women. Device implantation was achieved in over 99% of cases, with no difference between men and women.

**Figure 3:**
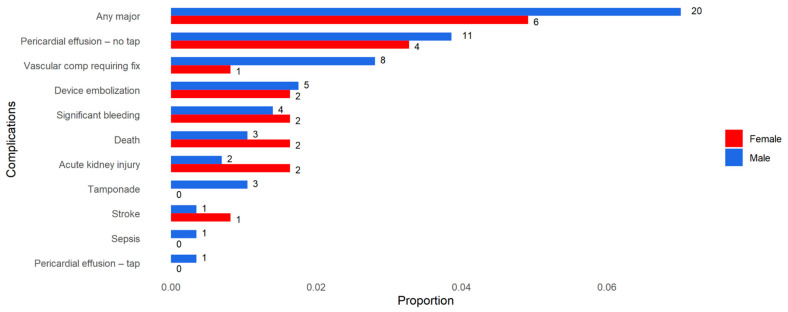
Procedure complications. Twenty-six (6.4%) patients had a major complication (not including untapped pericardial effusion, acute kidney injury and sepsis). Number of patients per complication per gender are presented. Men are in blue and women are in red.

Thirty-eight periprocedural complications were recorded, 33 of which were considered major complications affecting 26 patients (6.4%). There were no significant differences in periprocedural complication rates between men and women (men 7.0%, women 4.9%, *p* = 0.51). Periprocedural complications are listed in Table 2 and presented in Figure 3. The procedure success rate was 93.4%, with no difference between sexes (*p* = 0.485). In a univariate analysis for procedure success, we found no single predictive variable (Table S4 in the Supplementary Materials). The median hospitalization length was 1 day (range 0–30). The most common antithrombotic regimen recommended at discharge was dual antiplatelet therapy (DAPT) with Aspirin and Clopidogrel (72.7%), irrespective of sex (*p* = 0.67).

### Follow-Up

Initial post-procedural follow-up occurred at a median of 57 days post-procedure (IQR 43–88 days). Out of the 407 cases, follow-up imaging was reported for 339 patients (83.3%) with either CCTA (n = 92) or TEE (n = 247). The imaging demonstrated device thrombosis in 17 patients (5.2%) and any peri device leak in 81 patients (24.6%), with no difference in thrombosis or device leaks between men and women (*p* = 1.0). On first follow-up, antithrombotic therapy was modified in 319 patients (78.4%), most typically downgraded from DAPT to SAPT with either Aspirin (n = 160), Clopidogrel (n = 98), or no antithrombotic therapy (n = 19). No differences were noted in treatment regimens between sexes upon discharge (*p* = 0.66) or follow-up (*p* = 0.1) (Table 3).

Long-term follow-up (median 542 days IQR [237,1162]) was available for 379 patients (93.4%). Primary and secondary outcomes (crude data and Kaplan–Meier estimates) are summarized in Table 3.

**Figure 4:**
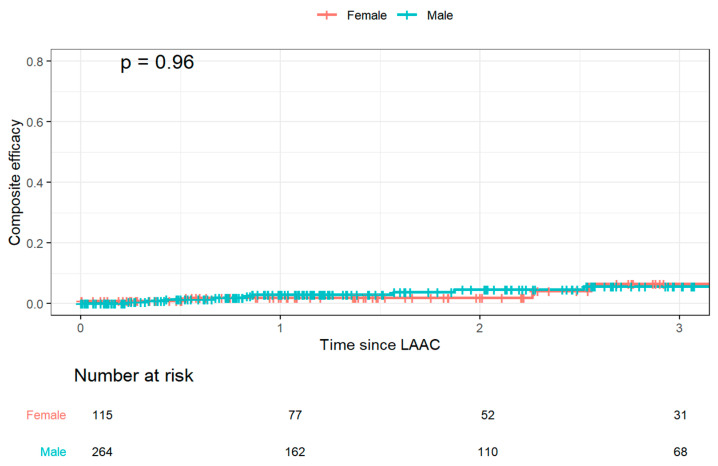
Kaplan–Meier plot for estimated composite efficacy (1 year ischemic stroke and systemic embolism) up to 3 years follow-up.

The KM estimate of the primary efficacy endpoint was 2.6 ± 0.9% at 1 year and 5.9 ± 1.7% at 3 years, with no significant difference between men and women (HR 1.03 (0.32, 3.36), *p* = 0.96) (Figure 4).

**Figure 5:**
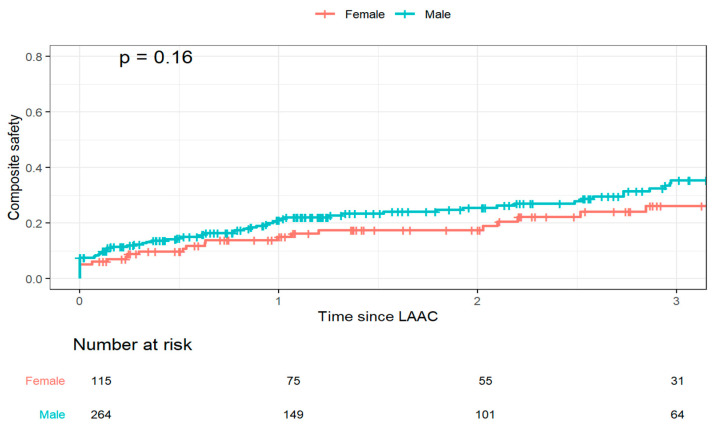
Kaplan–Meier plot for estimated composite safety (1 year all-cause mortality, procedural complications or major bleeding) up to 3 years follow-up.

The KM estimate of the primary safety endpoint was 19.2 ± 2.2% at 1 year and 32.6 ± 3.0% at 3 years, with no significant difference between men and women (HR 1.40 (0.87, 2.25), *p* = 0.16) (Figure 5).

The KM estimation of all-cause death at 1 year was 9.3 ± 1.6%, and 21.4 ± 2.9% at 3 years, with no significant difference between men and women (HR 1.55 (0.81, 2.96), *p* = 0.18) (Figure S1 in the Supplementary Materials).

The KM estimation of significant bleeding at 1 year was 8.9 ± 1.6%, and 14.7 ± 2.3% at 3 years, with no significant difference between men and women (HR 1.08 (0.55, 2.12), *p* = 0.82) (Figure S2 in the Supplementary Materials).

The KM estimation of the neurovascular event rate at 1 year was 3.6 ± 1.1%, and 7.8 ± 1.9% at 3 years, with no significant difference between men and women (HR 0.92 (0.35, 2.45), *p* = 0.87) (Figure S3 in the Supplementary Materials).

All primary and secondary endpoints were tested using a Cox proportional hazard model, adjusting for age, sex, CHADS2 and HAS-BLED scores, with no differences between men and women. Cox proportional hazard models are displayed in Table S5 in the Supplementary Materials.

To control for variance in baseline characteristics, two-stage propensity score-matching was performed with subsequent KM estimates of the primary efficacy and safety outcomes, as well as a Cox proportional hazard model. No differences between sexes were noted for this sub-analysis (HR 0.54 (0.25–8.78), *p* = 0.68 and HR 0.55 (0.74–2.67), *p* = 0.3, respectively, Figures S4–S6, Table S6 in the Supplementary Materials).

A subgroup analysis was performed for the primary efficacy and safety endpoints, including the age cutoff of 75 years, the device type (WM vs. AA) and lower BMI potential effect in women, with no differences noted between sexes (Figures S7 and S8 in the Supplementary Materials).

## 4. Discussion

### 4.1. Principal Findings

In this multicenter, real-world analysis from the Israeli LEADER registry, we evaluated sex-related differences in short- and long-term outcomes following left atrial appendage closure (LAAC), using both Watchman (2.5 and FLX) and Amplatzer Amulet devices. Among 407 consecutive patients with atrial fibrillation (AF) undergoing LAAC, women represented 30% of the cohort. Procedural success (defined as successful exclusion of the LAA without complications) exceeded 93% for both sexes, and peri-procedural complication rates were low and comparable between men and women. During a median follow-up of 18 months (IQR [8–38]), there were no significant sex differences in rates of ischemic stroke, systemic embolism, major bleeding, or all-cause mortality up to 3 years post-procedure.

Collectively, these findings contrast with earlier reports suggesting increased procedural risk among women, while reinforcing the equivalent long-term safety and efficacy of LAAC across sexes in contemporary practice.

### 4.2. Periprocedural Complications

Previous large registries have consistently reported higher peri-procedural complication rates among women undergoing LAAC. Analyses from the NCDR LAAO Registry, the Amulet IDE trial and the NIS (National Inpatient Sample) demonstrated that female sex independently predicted major adverse events, particularly pericardial effusion, vascular complications, major bleeding and in-hospital death [10,18,19]. Ismayl et al. stated that women were more susceptible to developing vascular complications after procedural interventions, owing to their complex vascular anatomy and smaller body size [20]. Several mechanisms have been proposed to explain these observations, including smaller atrial and vascular dimensions, thinner atrial walls, and differences in sheath-to-vessel ratios, relative to their body size [21]. In our cohort, however, the subgroup analysis revealed no sex-related differences in BMI among patients experiencing safety events (Figure S8 in the Supplementary Materials), suggesting that body habitus alone does not account for procedural risk in this population.

The overall major peri-procedural complication rate in the LEADER registry was 6.4%, in line with international benchmarks; this is slightly higher than the rates reported in the Amulet IDE (4.5%) and comparable to the overall rate in the SWISS-APERO study (9.0% AA vs. 2.7% WM-FLX) and the older PREVAIL/PROTECT-AF (4.2–8.7%) trials [22,23,24]. In contrast to the previously mentioned studies, our study demonstrated no significant sex differences in peri-procedural complications, ultimately showing no excess procedural risk among women (7.0% vs. 4.9%, *p* = 0.51).

With regard to device-specific differences, several studies comparing WM and AA devices revealed variations in the overall periprocedural outcomes between devices, while long-term outcomes remained similar [22,23,24,25]. In a previous study from the LEADER registry, we reported no significant difference in periprocedural complications between platforms [26].

When analyzing device-specific complications, no meaningful differences were noted between the Watchman and Amplatzer platforms for either sex (Figures S7 and S8 in the Supplementary Materials). Device-related thrombosis and the peri-device leak rates were comparable, and successful exclusion of the left atrial appendage was achieved irrespective of sex. These findings align with large multicenter studies analyzing device-specific sex differences, such as the Amulet IDE, which evaluated both AA and WM 2.5 devices, and the Italian FLX registry, which evaluated the newer generation Watchman FLX [15,18].

### 4.3. Long-Term Outcomes

Despite variability between studies and registries regarding peri-procedural outcomes, many of the studies mentioned thus far have shown similar long-term efficacy and safety outcomes for both men and women. Analyses including the EWOLUTION, LAARGE, Amulet IDE and Italian-FLX registries, while differing in the peri-procedural phases, all reported similar efficacy and safety outcomes in men and women up to 2–4 years post-implantation [13,15,18,27].

Long-term outcomes in the LEADER registry reaffirm the durability and safety of LAAC in both men and women. Over 3 years of follow-up, rates of ischemic stroke or systemic embolism (5.9%), major bleeding (14.7%), and all-cause mortality (21.4%) were comparable between sexes. These data align with contemporary evidence indicating that once the acute procedural phase is successfully navigated, long-term outcomes do not differ by sex. This holds true for thromboembolic prevention, bleeding risk reduction, and mortality. Importantly, device-related complications (e.g., device-related thrombus or peri device leak) were infrequent and did not vary between sexes, supporting the robust long-term performance of both device platforms in routine clinical use.

### 4.4. Limitations

This study has several limitations. First, as an observational registry, it is subject to inherent selection and reporting biases, despite prospective data collection. Second, although the study is multi-center, the overall sample size—particularly the number of women—is small and, as such, underpowered to detect small differences between groups (a potential for type II error). Third, follow-up imaging was not available for all patients, which may underestimate the incidence of device-related complications such as peri device leak or device-related thrombus. Furthermore, variance in follow-up imaging modality may introduce measurement bias as per differences in the sensitivity and specificity of detecting leaks and thrombi. Fourth, differences in antithrombotic regimens and procedural techniques between centers were not standardized, potentially influencing outcomes. Lastly, residual confounding by unmeasured variables cannot be excluded. Nevertheless, the LEADER registry represents one of the largest contemporary Israeli cohorts with comprehensive long-term follow-up, providing valuable real-world insight into sex-specific outcomes after LAAC.

### 4.5. Summary

This multicenter, real-world experience demonstrates that LAAC offers comparable procedural safety and long-term efficacy in men and women. In contrast to earlier reports suggesting excess peri-procedural risk in women, our findings indicate that with current procedural techniques, experienced operators, and modern device iterations, sex does not pose an independent determinant of procedural or clinical outcomes. These results reinforce the role of LAAC as an effective, nonpharmacologic stroke prevention strategy in AF patients of both sexes, provided that appropriate procedural planning and individualized follow-up are ensured.

## Figures and Tables

**Table 1:** Baseline characteristics and comorbidities.

Baseline Characteristics	All Patients	Women	Men	*p*
n	407	122	285	
Age, years (mean (SD))	77.27 (8.45)	76.91 (8.37)	77.42 (8.49)	0.574
Height, cm (mean (SD))	168.40 (8.80)	160.63 (7.36)	171.67 (7.16)	<0.001
Weight, Kg (mean (SD))	79.44 (15.64)	72.78 (15.58)	82.25 (14.82)	<0.001
BMI (mean (SD))	27.94 (4.74)	28.13 (5.27)	27.87 (4.51)	0.614
Creatinine, mg/dL (mean (SD))	1.24 (0.70)	1.08 (0.72)	1.30 (0.69)	0.009
GFR, MDRD (mean (SD))	64.05 (31.05)	69.70 (25.01)	61.81 (32.92)	0.036
Hgb, g/dL (mean (SD))	11.58 (2.30)	10.87 (2.36)	11.90 (2.21)	<0.001
CHADS2 (mean (SD))	3.11 (1.29)	3.06 (1.27)	3.13 (1.30)	0.622
CHADS_VASC (mean (SD))	5.10 (1.63)	4.95 (1.49)	5.17 (1.68)	0.217
HASBLED (mean (SD))	3.87 (0.95)	3.81 (0.91)	3.89 (0.96)	0.437
Comorbidities				
Diabetes Mellitus (%)	185 (45.6)	49 (40.2)	136 (47.9)	0.186
PVD (%)	81 (20.0)	27 (22.3)	54 (19.1)	0.543
Liver disease (%)	10 (5.0)	4 (6.9)	6 (4.2)	0.66
Recurrent Falls (%)	37 (9.2)	14 (11.6)	23 (8.2)	0.368
Active malignancy (%)	42 (10.3)	11 (9.0)	31 (10.9)	0.698
Chronic lung disease (%)	31 (15.6)	5 (8.6)	26 (18.4)	0.128
Coronary heart disease (%)	193 (47.7)	40 (33.1)	153 (53.9)	<0.001
Valvular heart disease (%)	70 (18.1)	19 (16.4)	51 (18.9)	0.658
Congestive heart failure (%)	117 (28.9)	29 (24.0)	88 (31.0)	0.191
Preserved/Good LV function (LVEF ≥ 50%)	299 (73.5%)	106 (89.8%)	193 (70.2%)	0.001
Mild LV dysfunction (LVEF 45–49%)	24 (5.9%)	4 (3.4%)	20 (7.3%)	
Mild-mod LV dysfunction (LVEF 40–44%)	15 (3.7%)	2 (1.7%)	13 (4.7%)	
Moderate LV dysfunction (LVEF 35–39%)	23 (5.7%)	4 (3.4%)	19 (6.9%)	
Mod-severe LV dysfunction (LVEF 30–34%)	9 (2.2%)	1 (0.8%)	8 (2.9%)	
Severe LV dysfunction (LVEF ≤ 30%)	23 (5.7%)	1 (0.8%)	22 (8%	
Cerebrovascular disease (%)	227 (56.2)	69 (57.0)	158 (55.8)	0.911
Dementia—moderate (%)	6 (1.6)	3 (2.7)	3 (1.1)	0.3674
HTN (%)	344 (86.0)	103 (85.8)	241 (86.1)	1
Previous significant bleeding (%)	310 (84.7)	89 (80.9)	221 (86.3)	0.245
Pre-procedure imaging				
Pre-procedure TEE (%)	149 (74.9)	40 (70.2)	109 (76.8)	0.431
Pre-procedure CCTA (%)	238 (67.0)	77 (68.8)	161 (66.3)	0.731

**Table 2:** Peri-procedural complications.

Complications	All Patients	Women	Men	*p*
n	407	122	285	
Procedural success with no peri-procedural complications (%)	380 (93.4%)	116 (95.1%)	264 (92.6%)	0.485
Pericardial effusion—not requiring tap (%)	15 (3.7)	4 (3.3)	11 (3.9)	1
Pericardial effusion—requiring tap (%)	1 (0.2)	0 (0.0)	1 (0.4)	1
Tamponade (%)	3 (0.7)	0 (0.0)	3 (1.1)	0.614
Vascular complication requiring fix (%)	9 (2.2)	1 (0.8)	8 (2.8)	0.378
Device embolization (%)	7 (1.7)	2 (1.6)	5 (1.8)	1
Significant bleeding (%)	6 (1.5)	2 (1.6)	4 (1.4)	1
Death (%)	5 (1.2)	2 (1.6)	3 (1.1)	0.999
CVA (%)	2 (0.5)	1 (0.8)	1 (0.4)	1
AKI (%)	4 (1.0)	2 (1.6)	2 (0.7)	0.741
Sepsis (%)	1 (0.2)	0 (0.0)	1 (0.4)	1
Major complications * (%)	26 (6.4)	6 (4.9)	20 (7.0)	0.512

* Refers to pericardial effusion requiring tap, tamponade, vascular complication requiring fix, device embolization, significant bleeding, CVA and death.

**Table 3:** Long-term outcomes. Displaying crude data, Kaplan–Meier estimates, treatment at discharge and follow-up treatment.

Outcomes	All Patients	Women	Men	*p*
n	407	122	285	
Crude data				
Follow-up TEE (%)	247 (64.0)	71 (60.7)	176 (65.4)	0.437
Follow-up CCTA (%)	92 (23.1)	28 (23.7)	64 (22.9)	0.954
Device thrombosis (%)	17 (5.2)	5 (5.1)	12 (5.2)	1
Device leak (%)	81 (24.6)	24 (24.5)	57 (24.7)	1
1-year mortality (%)	30 (7.4)	8 (6.6)	22 (7.7)	0.831
3-year mortality (%)	52 (12.8)	12 (9.8)	40 (14.1)	0.311
1-year ischemic stroke (%)	8 (2.0)	2 (1.7)	6 (2.1)	1
3-year ischemic stroke (%)	13 (3.2)	4 (3.3)	9 (3.2)	1
1-year neurovascular event * (%)	11 (2.7)	3 (2.5)	8 (2.8)	1
3-year neurovascular event * (%)	18 (4.4)	6 (5.0)	12 (4.2)	0.943
1-year bleeding (%)	30 (7.5)	7 (5.8)	23 (8.2)	0.546
3-year bleeding (%)	40 (10.0)	12 (10.0)	28 (9.9)	1
1-year efficacy outcome (%)	8 (2.0)	2 (1.7)	6 (2.1)	1
3-year efficacy outcome (%)	13 (3.2)	4 (3.3)	9 (3.2)	1
1-year safety outcome (%)	66 (16.5)	16 (13.3)	50 (17.8)	0.339
3-year safety outcome (%)	91 (22.7)	23 (19.2)	68 (24.2)	0.331
Kaplan–Meier estimates for long-term outcomes				
	% (SD)	% (SD)	% (SD)	
Composite efficacy estimate at 1 year (%)	2.6 (0.9)	1.9 (1.4)	3.0 (1.2)	0.96
Composite efficacy estimate at 3 years (%)	5.9 (1.7)	6.5 (3.4)	5.7 (2.0)	
Composite safety estimate at 1 year (%)	19.2 (2.2)	15.1 (3.5)	21.0 (2.7)	0.16
Composite safety estimate at 3 years (%)	32.6 (3.0)	26.1 (5.0)	35.5 (3.9)	
Death rate estimate at 1 year (%)	9.3 (1.6)	7.9 (2.7)	9.9 (2.0)	0.18
Death rate estimate at 3 years (%)	21.4 (2.9)	14.9 (4.3)	24.2 (3.6)	
Significant bleeding rate estimate at 1 year (%)	8.9 (1.6)	6.7 (2.5)	10.0 (2.0)	0.82
Significant bleeding rate estimate at 3 years (%)	14.7 (2.3)	14.7 (4.2)	14.6 (2.8)	
Neurovascular event rate estimate at 1 year (%)	3.6 (1.0)	3.1 (1.8)	3.8 (1.3)	0.87
Neurovascular event rate estimate at 3 years (%)	7.8 (1.9)	8.2 (3.7)	7.4 (2.2)	
Treatment at discharge				0.66
SAPT	63 (15.5)	18 (14.8)	45 (15.8)	
DAPT	296 (72.7)	87 (71.3)	209 (73.3)	
OAC	33 (8.1)	13 (10.7)	20 (7.0)	
Other	15 (3.7)	4 (3.3)	11 (3.9)	
Treatment at follow-up				0.10
SAPT	258 (63.4)	79 (64.8)	179 (62.8)	
DAPT	84 (20.6)	18 (14.8)	66 (23.2)	
OAC	32 (7.9)	14 (11.5)	18 (6.3)	
None	19 (4.7)	8 (6.6)	11 (3.9)	
Other	14 (3.4)	3 (2.5)	11 (3.9)	

* Neurovascular events = ischemic stroke + hemorrhagic stroke.

## Data Availability

Data pertaining to this manuscript will be made available upon reasonable request from the corresponding author.

## Supplementary Materials

The following supporting information can be downloaded at: https://www.mdpi.com/article/10.3390/jcm15041604/s1, Table S1: LAAC procedure temporal distribution throughout the study period at 4 medical centers; Table S2: Platform generation distribution between sexes; Table S3: LAAC indications divided by gender; Table S4: Univariate analysis for procedure success; Figure S1: Kaplan-Meier plot for estimated death rate up to 3 years follow-up. Probabilities were estimated using the Kaplan–Meier method. Hazard ratios for crude and adjusted death rates were calculated using Cox proportional hazards models; Figure S2: Kaplan-Meier plot for estimated bleeding events (Intracranial and intracerebral, GI/GU, fatal bleeding ) up to 3 years follow-up. Probabilities were estimated using the Kaplan–Meier method. Hazard ratios for crude and adjusted bleeding events were calculated using Cox proportional hazards models; Figure S3: Kaplan-Meier plot for estimated neurovascular events (Ischemic & hemorrhagic stroke) up to 3 years follow-up. Probabilities were estimated using the Kaplan–Meier method. Hazard ratios for crude and adjusted neurovascular events were calculated using Cox proportional hazards models; Table S5: Cox proportional hazards model for long term outcomes - Men vs. Women. Hazard ratios with 95% confidence interval; Figure S4: Love plot displaying the balance of covariates before and after propensity score matching (PSM); Figure S5: KM estimate of the primary efficacy endpoint for the matched cohort after PSM. Probabilities were estimated using the Kaplan–Meier method. Hazard ratios were calculated using Cox proportional hazards models; Figure S6: KM estimate of the primary safety endpoint for the matched cohort after PSM. Probabilities were estimated using the Kaplan–Meier method. Hazard ratios were calculated using Cox proportional hazards models; Table S6: PSM cohort- Cox proportional hazards model for long term outcomes - Men vs. Women. Hazard ratios with 95% confidence interval; Figure S7: Subgroup analysis for the primary efficacy outcome. Figure S8: Subgroup analysis for the primary safety outcome.
