# Supplementary material for: Sex Differences in Long-Term Outcomes of Left Atrial Appendage Closure—Analysis from the LEADER Registry
Source: J Clin Med. 2026 Feb 19;15(4):1604. doi: 10.3390/jcm15041604 (PMC12941582; doi:10.3390/jcm15041604)
Supplement: Supplementary file 1 [file jcm-15-01604-s001.zip › jcm-4081193-supplementary.pdf]

Supplementary

Hospital/Year	2010	2011	2012	2013	2014	2015	2016	2017	2018	2019	2020	2021	2022	2023
Beilinson	0	0	0	9	7	8	8	6	8	8	6	14	20	27
Sheba	9	15	17	16	4	13	20	16	31	38	26	0	0	0
Ichilov	0	0	0	0	0	0	0	0	25	19	17	5	1	0
Meir	0	0	0	0	0	0	0	0	0	0	10	4	0	0

Table S1. LAAC procedure temporal distribution throughout the study period at 4 medical centers.

Device type	# of devices total (%)	# of devices men (%)	# of devices women (%)	p_value
WM 2.5	74	48 (16.8%)	26 (21.3%)	0.11
WM FLX	94	70 (24.6%)	24 (19.7%)	
Amplatzer Cardiac Plug 1	34	26 (9.1%)	8 (6.6%)	
Amplatzer Amulate	205	141 (49.5%)	64 (52.5%)	

Table S2. Platform generation distribution between sexes.

Indication	Total	Women	Men	p_value
GI/GU bleeding	162 (39.8%)	54 (44.3%)	108 (37.9%)	0.17
Intracerebral/Intracranial Hemorrhage	148 (36.4%)	41 (33.6%)	107 (37.5%)	
Other	47 (11.5%)	8 (6.6%)	39 (13.7%)	
Recurrent falls	13 (3.2%)	4 (3.3%)	9 (3.2%)	

DOAC failure	10 (2.5%)	6 (4.9%)	4 (1.4%)	
Anemia	9 (2.2%)	3 (2.5%)	6 (2.1%)	
MRI high ICH risk	7 (1.7%)	1 (0.8%)	6 (2.1%)	
Hemorrhagic transformation	5 (1.2%)	3 (2.5%)	2 (0.7%)	
Missing	4 (1%)	1 (0.8%)	3 (1.1%)	
Patient preference	2 (0.5%)	1 (0.8%)	1 (0.4%)	

Table S3. LAAC indications divided by gender.

Predictor	OR	95 CI	P value
Device type	0.98	0.43–2.15	0.96
Sex	0.65	0.23-1.56	0.36
Age	0.99	0.94–1.03	0.61
CHADSVASC	0.83	0.65-1.06	0.13
Diabetes Mellitus	0.9	0.41-2	0.80
HASBLED	0.83	0.53-1.26	0.83
GFR	0.69	0.22-3.04	0.69
LVEF	1.67	0.33-30.54	0.62

Table S4. Univariate analysis for procedure success.

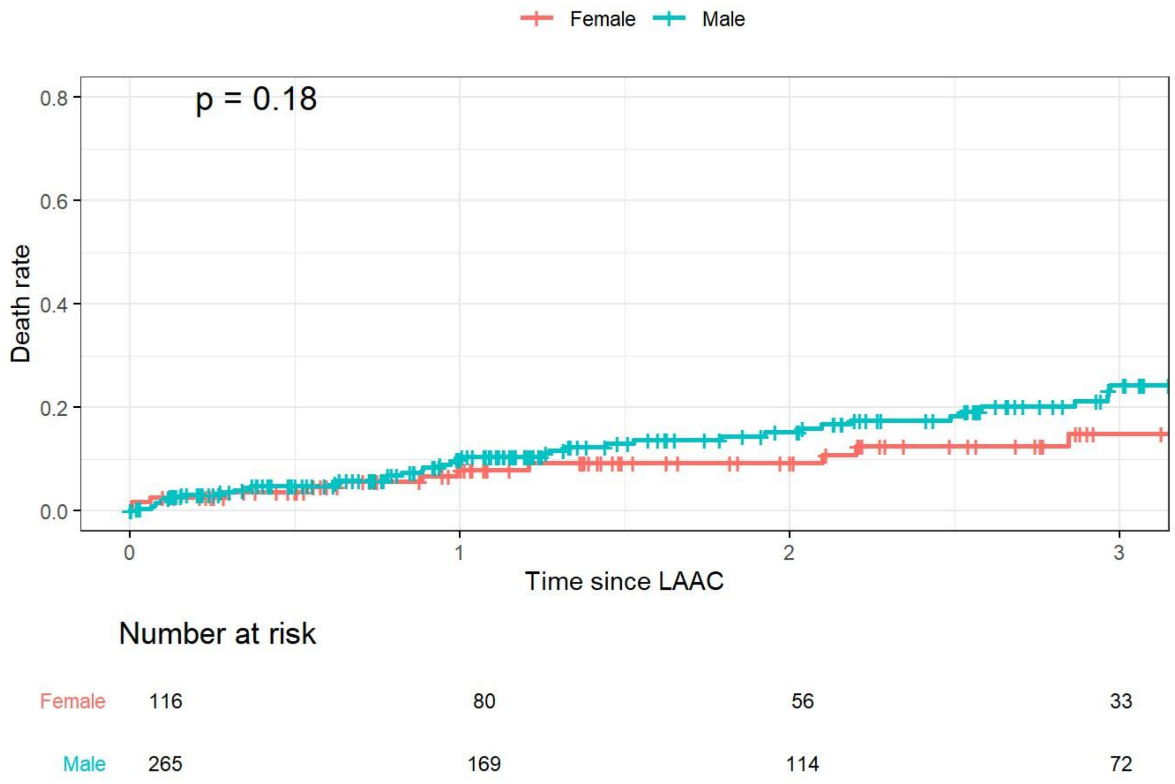

Figure S1. Kaplan–Meier plot for estimated death rate up to 3 years follow-up. Probabilities were estimated using the Kaplan–Meier method. Hazard ratios for crude and adjusted death rates were calculated using Cox proportional hazard models.

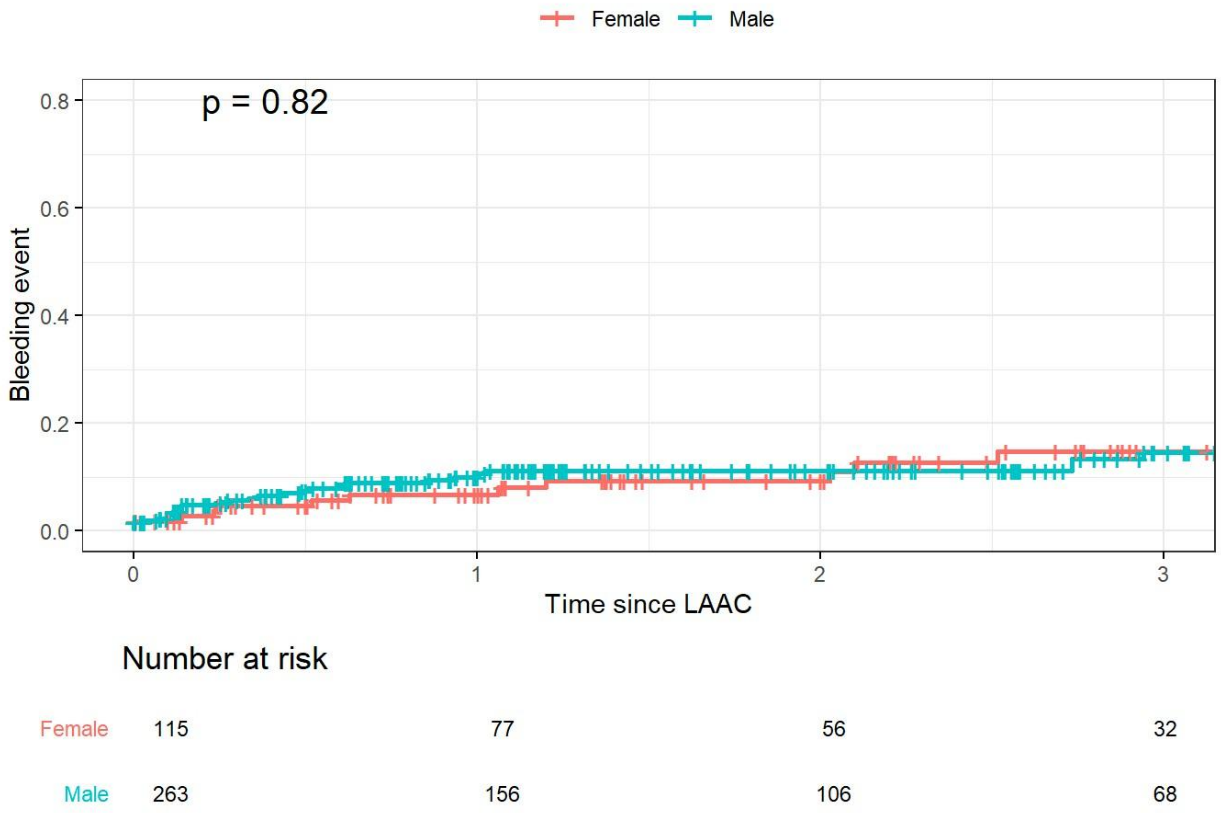

Figure S2. Kaplan–Meier plot for estimated bleeding events (intracranial and intracerebral, GI/GU, fatal bleeding) up to 3 years follow-up.
Probabilities were estimated using the Kaplan–Meier method. Hazard ratios for crude and adjusted bleeding events were calculated using Cox proportional hazard models.

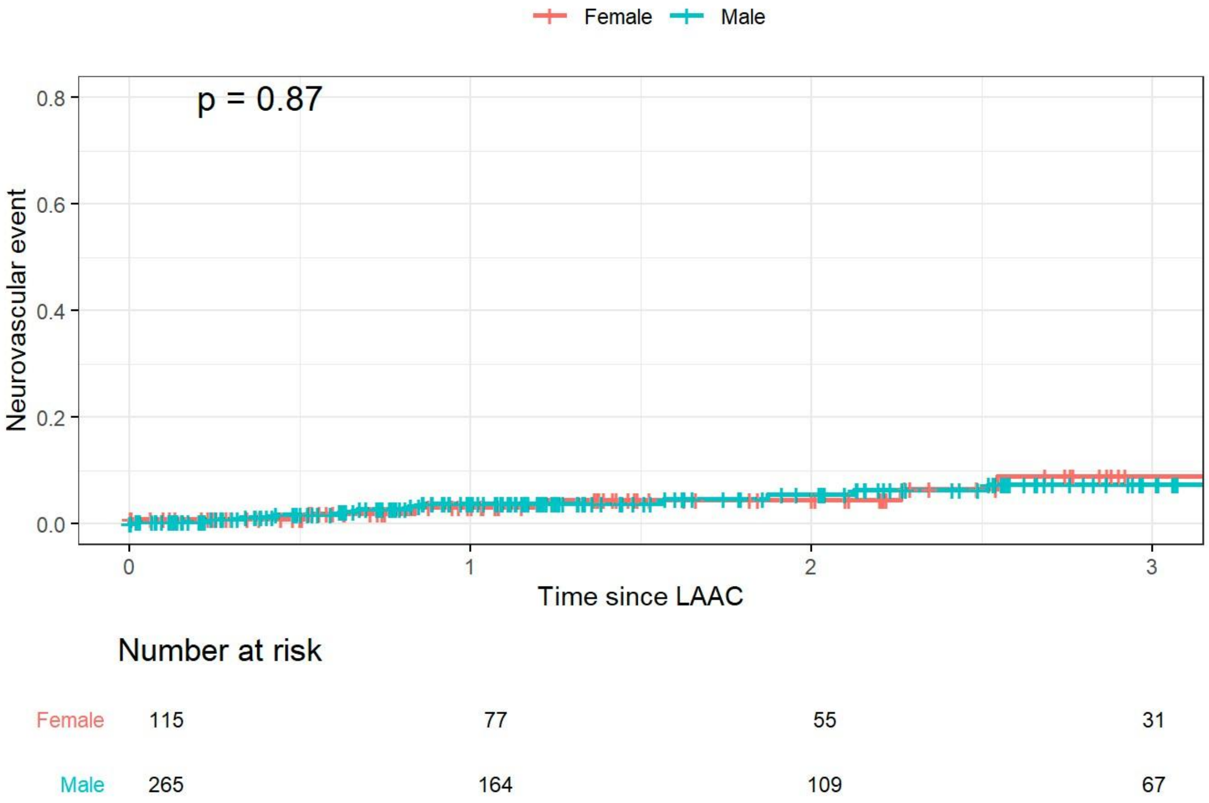

Figure S3. Kaplan–Meier plot for estimated neurovascular events (ischemic and hemorrhagic stroke) up to 3 years follow-up.
Probabilities were estimated using the Kaplan–Meier method. Hazard ratios for crude and adjusted neurovascular events were calculated using Cox proportional hazard models.

Outcome	n	Crude model HR (CI)	P value	Adjusted model HR (CI)	P value
1-year mortality	381	1.25 (0.55, 2.80)	0.59	1.0 (0.44, 2.30)	0.99
3-year mortality	381	1.55 (0.81, 2.96)	0.18	1.34 (0.69, 2.58)	0.39
1-year major bleeding	378	1.49 (0.64, 3.47)	0.36	1.44 (0.62, 3.38)	0.4
3-year major bleeding	378	1.08 (0.55, 2.12)	0.82	1.05 (0.53, 2.07)	0.89
1-year neurovascular outcome	380	1.19 (0.32, 4.48)	0.8	1.12 (0.29, 4.27)	0.87

3-year neurovascular outcome	380	0.92 (0.35, 2.45)	0.87	0.84 (0.31, 2.26)	0.73
1-year safety outcome	379	1.44 (0.82, 2.52)	0.2	1.34 (0.76, 2.36)	0.32
3-year safety outcome	379	1.40 (0.87, 2.25)	0.16	1.28 (0.79, 2.07)	0.31
1-year efficacy outcome	379	1.36 (0.27, 6.73)	0.71	1.33 (0.26, 6.66)	0.73
3-year efficacy outcome	379	1.03 (0.32, 3.36)	0.96	1.02 (0.54, 1.94)	0.93
Models adjusted for age, sex, chads_vasc and has_bled scores					

Table S5. Cox proportional hazard model for long term outcomes—men vs. women.
Hazard ratios with 95% confidence interval.

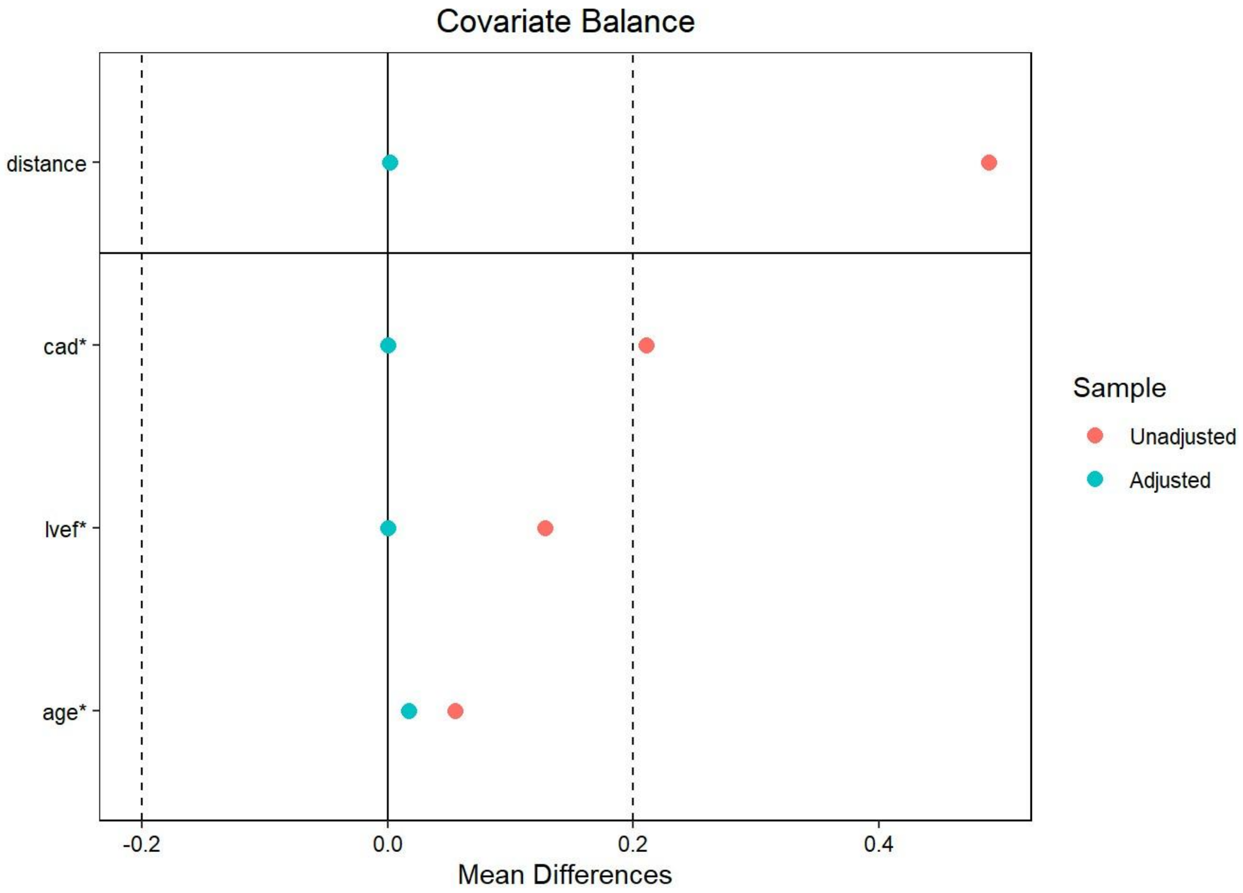

Figure S4. Love plot displaying the balance of covariates before and after propensity score-matching (PSM).

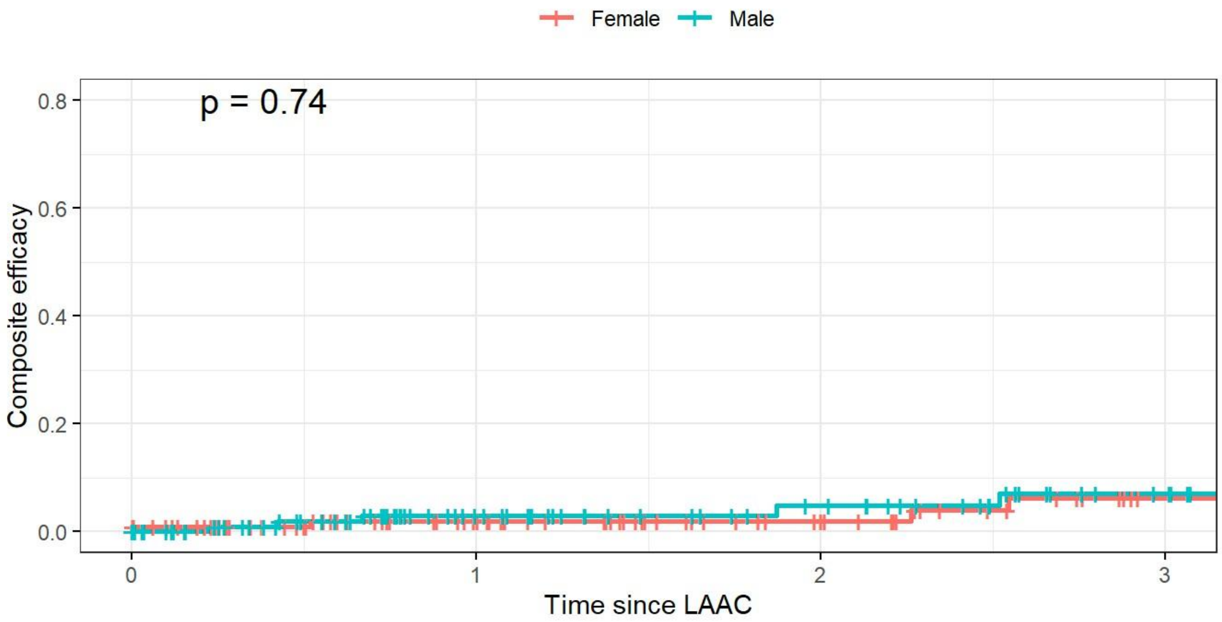

Figure S5. KM estimate of the primary efficacy endpoint for the matched cohort after PSM. Probabilities were estimated using the Kaplan–Meier method. Hazard ratios were calculated using Cox proportional hazard models.

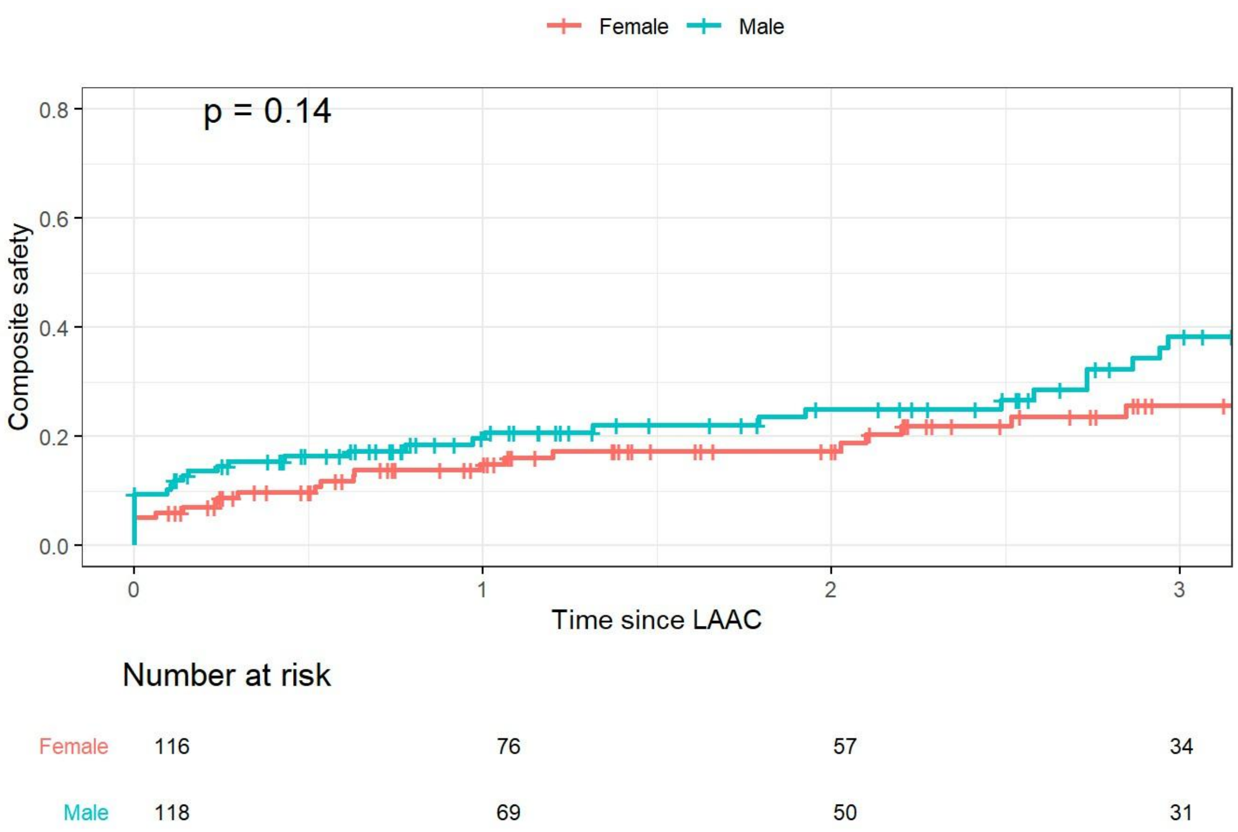

Figure S6. KM estimate of the primary safety endpoint for the matched cohort after PSM. Probabilities were estimated using the Kaplan–Meier method. Hazard ratios were calculated using Cox proportional hazard models.

Outcome	Hazard ratio (CI)	P value
1-year efficacy outcome	0.54 (0.25-8.78)	0.68
1-year safety outcome	0.55 (0.74-2.67)	0.3

Table S6. PSM cohort: Cox proportional hazard model for long term outcomes—Men vs. Women.
Hazard ratios with 95% confidence interval.

Forest 1 – Efficacy @ 3 years

Subgroup	HR (95% CI)	P-int
Age: <75	0.66 (0.11-3.92)	
Age: ≥75	1.36 (0.27-6.74)	0.528
Device Type: AA	2.11 (0.25-18.11)	
Device Type: WM	0.62 (0.14-2.76)	0.358
CHA2DS2-VASc: High	0.78 (0.19-3.11)	
CHA2DS2-VASc: Low	1.61 (0.17-15.43)	0.596
HAS-BLED: High	0.83 (0.21-3.31)	
HAS-BLED: Low	1.45 (0.15-14.04)	0.660
BMI: 20-27	1.22 (0.25-6.06)	
BMI: >27	0.69 (0.12-4.12)	1.000

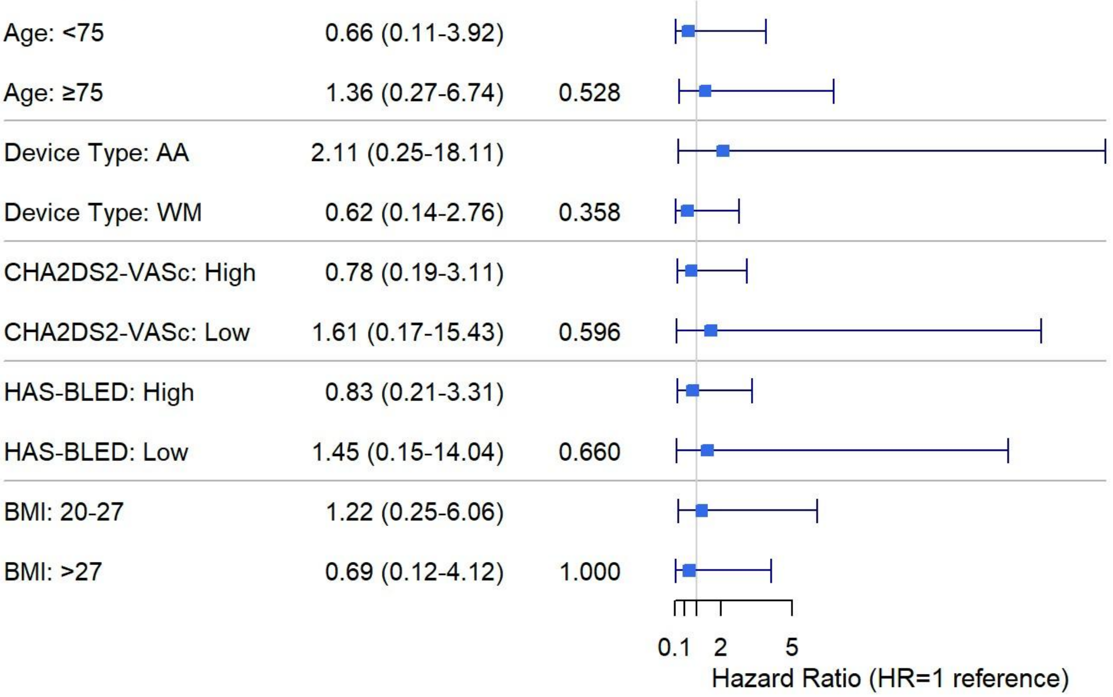

Figure S7. Subgroup analysis for the primary efficacy outcome.

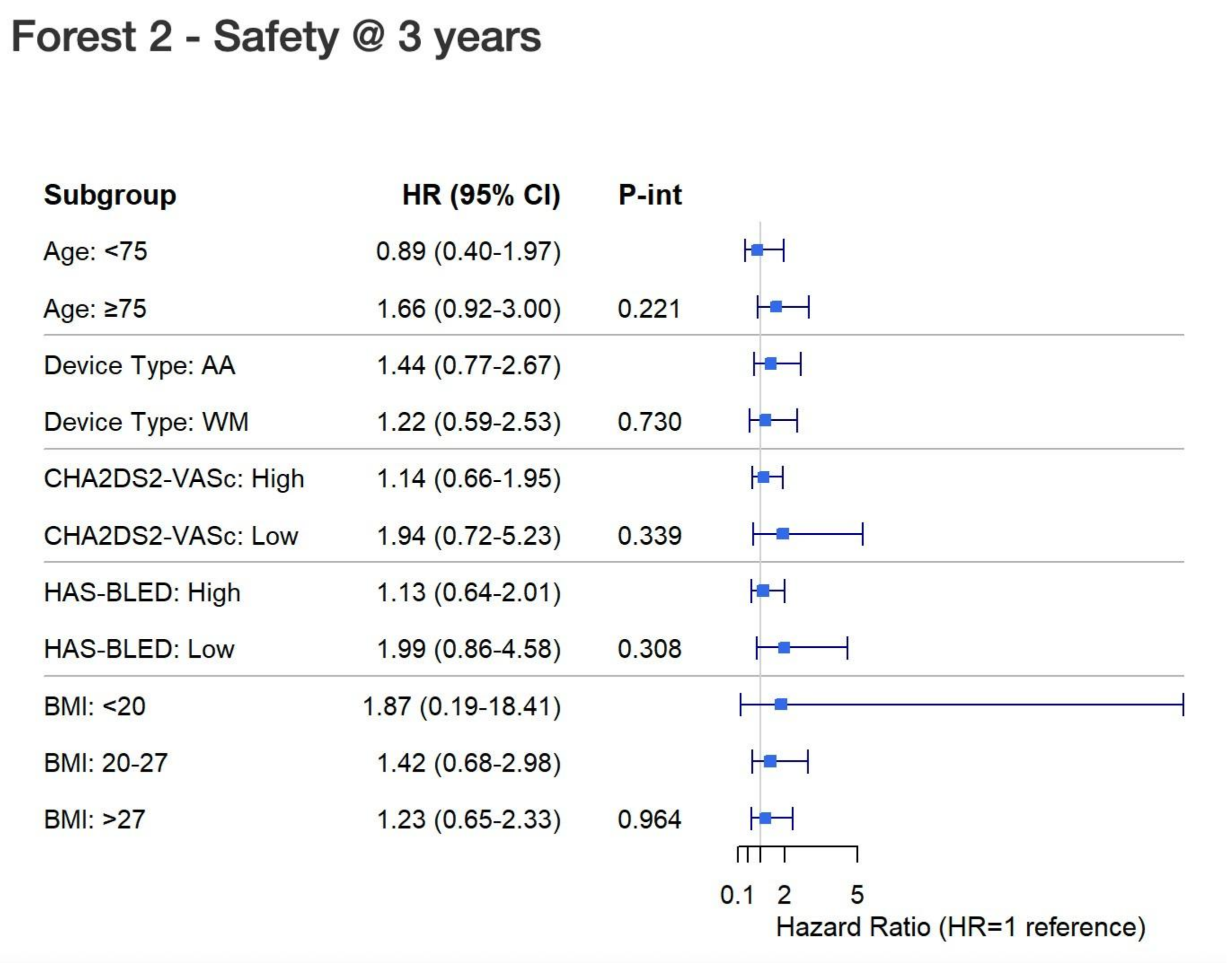

Figure S8. Subgroup analysis for the primary safety outcome.
